# Supplementary material for: Estimation of the firing behaviour of a complete motoneuron pool by combining electromyography signal decomposition and realistic motoneuron modelling
Source: PLoS Comput Biol. 2022 Sep 29;18(9):e1010556. doi: 10.1371/journal.pcbi.1010556 (PMC9553065; doi:10.1371/journal.pcbi.1010556)
Supplement: Attachment — Submitted filename: PCOMPBIOL-D-22-00412.pdf [file pcbi.1010556.s001.pdf]

# PCOMPBIOL-D-22-00412

## Estimation of the firing behaviour of a complete motoneuron pool by combining electromyography signal decomposition and realistic motoneuron modelling

Thank you for the opportunity to read and comment on this interesting manuscript. The manuscript presents a modeling framework that combines current “best practise” empirical data collection (decomposition of high-density grid surface electromyography, HDEMG, into discrete spike trains) with a population model of leaky-integrate-&-fire (LIF) neurons. The main contribution is to use the empirical data to constrain parameter choices in the model, and iteratively adjust the model to capture the dynamics of the empirical spike train data. The workflow for this process is new and a unique contribution to the field of modeling in this specialized domain of motor unit physiology. The authors use data from four previous publications to demonstrate a 4-step workflow (Figure 1 and 11) that includes some important validation components that I do not think have been attempted prior to this: 1) the empirical data from individual neurons are assigned rank orders within the population model; 2) the effective neural drive to the pool is calculated from the empirical data; and 3) the predicted discharge statistics from the model are compared to the empirical neuron discharges. The manuscript represents a substantial amount of work and there are many insights to be gained from a careful reading. I offer some general feedback for consideration to strengthen the manuscript and some specific comments below.

# General comments

1. “Computational code is available under request to the authors.” *PLOS Computational Biology* https://journals.plos.org/ploscompbiol/s/submission-guidelines#loc-software-submissions The submission guidelines for PLOS Comp Biology explicitly state that software “must be downloadable anonymously in sourse code form”, but the manuscript states interested parties must make a request to the authors. My prior reviews for this journal have included links to software on GitHub and I was able to run the modeling software myself during the review process. This permitted a more complete assessment of the PLOS goal that the software “be widely adopted by a broad community of users”. (my paraphrase from the software guidelines) I think the importance of this criteria should be met prior to publication in PLOS Computational Biology and reviewers should have the opportunity to use the software prior to publication. (Of course, I leave this to the discretion of the Editorial staff.)

2. Validation: The manuscript points out two ways in which the modeling workflow was validated (beginning on line 616). First, simulated spike trains are compared to the

rank-ordered empirical spike trains that are used to parameterize the model (recognizing the Nr-1 permutations). This is an important step the metrics demonstrate good agreement between the four sets of empirical data and the simulations. However, a key step is missing in this process. All the empirical data that is used to parameterize the model comes from a very specific experimental protocol, isometric trapezoidal force profiles (Fig 2). This is a very useful protocol for data collection, but has low external validity. The manuscript does not provide any evidence that the modeling workflow and parameters generated by fitting to the trapezoidal force profile would generalize to another contraction profile. This would be a very important step to show that the parameters of the model fit in one specific case, generalize to a different contraction trajectory.

3. Line 400 Sensitivity analysis - Cm: I have a number of issues with this section. First, specific membrane capacitance is a biophysical property that can be directly measured in a range of tissues (e.g. for measuring excocytosis https://www.pnas.org/doi/pdf/10.1073/pnas.79.21.6712, or in blood cells https://doi.org/10.1098/rsif.2017.0717 ) . It is not a free parameter and the range quoted on 405 is not accurate. Perhaps most relevant to the current manuscript, Cm is a ‘biological constant’ https://doi.org/10.1016/S0006-3495(00)76293-X and would best be treated as a fixed value for a pool of motor neurons. The results of fitting Cm (starting line 694) give the expected values, but really add nothing to the value of the manuscript overall. I would suggest removing these sections along with the figures that are based on alterations of specific membrane capacitance (e.g. Figure 14 & 15). A subsequent benefit of removing this material is that it shortens the overall manuscript making it more appealing to readers.

4. Sensitivity analysis - *suggestion*: I think a parameter that would be more interesting to explore in a sensitivity analysis is the number of motor units in the model. The section starting on line 204 indicates that estimates of the numbers of motor units vary from less than 200 to more than 400, and the latter value is chosen. In the anatomical estimate (Feinstein et al. 1955) the authors used old estimates of the proportion of large diameter myelinated axons that were thought to be afferent versus efferent; they assumed 40% of the fibers were afferent and 60% efferent. However, more recent work in the upper limb using improved methods (https://doi.org/10.1002/ana.25018, for which Dario Farina is a contributing author) estimate “sensory axons outnumber motor axons by a ratio of at least 9:1”. In Feinstein et al the number of motor axons was estimated as 445 assuming 60% efferents. Recalculation using the 9:1 ratio would give an estimate of about 75 motor units in TA. I think a sensitivity analysis that looked at numbers of motor units ranging from 100 - 400 may have been more interesting compared to the work on specific membrane capacitance mentioned above.

5. Line 252 I(t): I liked the care and attention to determining input current to the pool of motor neurons that has empirical origins in the decomposed spike trains. However, the use of current is problematic since synaptic input is a conductance not a current source.

That means as synaptic input increases, the apparent input resistance of the cell decreases and this can give rise to saturation (e.g. https://doi.org/10.1152/jn.00850.2016). Choosing to model this as synaptic conductance would have effects on the IP parameter (Line 322) and may circumvent the need to create 'saturation' (line 339) the way it is currently handled. This is a significant limitation (for this reviewer) that could be easily handled by using conductance synaptic input (e.g. as reviewed in https://doi.org/10.1007/s00422-006-0068-6). Attention to this detail seems to be more consistent with the attention to detail in the calculation of effective neural drive ($eND$).

6. MN-driven muscle model (starting at Line 724): I think that the choice of a Hill-type muscle model (described starting at line 427) is fine for a first iteration. When I read the text and examined Figure 16, I wondered if this step really added to the overall goal of the manuscript. I recognize that adding this was intended to demonstrate an application to a broader potential user base that measures isometric force. My intuition is that comparing the red lines to green lines in Fig 16 may just open the manuscript to criticism given the clear discrepancy in frequency content. Perhaps just the comparison of the blue line to the green is sufficient and an extension to force could come later, combined with models of surface EMG of the single units to compare force and sEMG signals as was done those many years ago in [29].

# Minor comments

- Page 11, lines 225-227: This was the first instance where in text citations changed from numbers to author name and year of publication. I wasn't sure if this change was intentional.
- Line 477, Figure 4B: I think the intention in this figure is to show that the data are not well fit to y = x (aka the line of identity). That isn't immediately clear when looking at the plot because the line of identity is not shown. The regression line (slope 0.9) is fine, but that doesn't seem to be the point of this figure.
- I realize that PLOS Computational Biology does not include the guidance to number equations. My preference would be to include numbers, simply because I was doing it myself on my copy of the manuscript. This is a minor issue that I leave to the authors and editors discretion.
- Figure 10 C: The presentation of a raster of spike trains is okay, but it might be nice to show instantaneous discharge rate (aka onion skin, e.g. https://doi.org/10.1016/j.jbiomech.2014.12.003) for readers familiar with this way of examining the output behavior of a pool of motor neurons.
- Discussion (Limitations, starting Line 871): This was a very extensive section in the discussion and I had to read over it a few times to understand how many discrete instances of limitations were being discussed. Perhaps this could be structurally improved by simply listing in the opening paragraph that X number of limitations will be discussed and then using explicit subheadings: *Limitation 1*, etc. I thought that perhaps

this section was written to preempt reviewers comments and became quite extensive. I think this section could be shortened.

- Discussion (Line 987 onwards): As stated in general comment #6, I am not sure if the addition of the muscle model helps or hinders the presentation of the framework for developing a pool of spiking neurons to compare with data from HDEMG. This section ends the entire manuscript and as I read it I seemed to be distracted more and more from the key contributions of the paper – rather than reinforcing the important insights gained. I think this could be dropped and replaced with a Conclusion section.
