# Supplementary material for: Estimation of the firing behaviour of a complete motoneuron pool by combining electromyography signal decomposition and realistic motoneuron modelling
Source: PLoS Comput Biol. 2022 Sep 29;18(9):e1010556. doi: 10.1371/journal.pcbi.1010556 (PMC9553065; doi:10.1371/journal.pcbi.1010556)
Supplement: Attachment — Submitted filename: Reviewers_comments_Final.docx [file pcbi.1010556.s002.docx]

Answers to the reviewers' comments

**Reviewer 1: Randall Powers**

This study describes the use of a set of leaky integrate-and-fire (LIF)motoneuron models to predict the recruitment and firing behavior of the complete pool of motoneurons (MNs) innervating a muscle, based on experimental recordings of a subset of MN spike trains during a trapezoidal isometric contraction. The authors demonstrate that the parameters of their LIF models can be tuned to recreate the firing behavior of the recorded MNs, and can also be used to predict the firing behavior of the entire pool. Using the activation of MNs from the entire pool as an input to a muscle model gives a much better fit to the normalized torque recorded during the contractions than does the input from just the recorded spike trains. This approach can thus help experimentalists and modelers better understand the relation between force production and the MN drive to the muscle. However, the approach does not provide much insight into the biophysical mechanisms controlling MN firing rate behavior given the many differences between a single-compartment model with no active conductances and real motoneurons with a variety of active conductances and an extended dendritic tree.

**Introductory comment**

We thank the reviewer for this clear summary of our work and its potential impacts for better understanding the relation between the CNS and muscle force generation. We also thank the reviewer for his detailed comments which helped us strengthening and clarifying the manuscript and in addressing the limitations of the considered single-compartment LIF model. We took care in addressing and answering each of the comments below, systematically referring to the lines of the revised version of the manuscript where changes were made. The changes are highlighted in turquoise in the new manuscript.

We wanted to mention to the reviewer that the code used to produce the results presented in the study will be made open-source, in agreement with the journal’s policy. It can be cloned for the still private Github repository at this url: **https://gitfront.io/r/AHDC/MApvQpDP4YJc/Caillet-et-al-2022-PLOS-Comput-Biol/** . The instructions on how to setup the ad hoc virtual environment necessary to run the scripts without prompting errors, and the description of the aims and contents of the scripts are provided in the repository README file.

It must be noted that all the simulations were re-run for this resubmission. This was necessary to address the reviewers’ comments and specifically for two reasons associated to the comments. First, $C_{m}$ is now not calibrated anymore in the $\left[ t_{r_{0}};t_{r_{3}} \right]$ range but set to a constant value in all trials, following the reviewer’s comments. Second, the values of the $R,C$ and $\tau$ parameters, based on the mathematical relationships provided in a preprint and now published manuscript (*Caillet, A. H., Phillips, A. T., Farina, D., & Modenese, L. (2022). Mathematical relationships between spinal motoneuron properties. eLife, 11, e76489*), were slightly modified following the reviewing process of that previous manuscript. With these modifications, in the current revised manuscript, the model predictions are strongly in line with the previous results, and overall provide more accurate predictions for Validations 1 and 2. Where necessary, the manuscript text was adapted to reflect the changes.

In line with the reviewer’s introductory summary, most of the reviewer’s comments focused on the limitations of the LIF modelling approach taken in this study. To provide the clearest possible answer to the reviewer’s comments, we regrouped the comments by topic.

**LIF model-related comments**

Line 282. The common synaptic current I(t) that drives spike generation is the total dendritic membrane current that arises not just from the net excitatory synaptic influx but also from intrinsic current generated by voltage-dependent Na and Ca channels.

We thank the reviewer for this very important comment as the definition of $I\left( t \right)$ in the previous version of the manuscript may have been a source of confusion for the reader. We now detail (L.140-143 & L.279-287) that the total dendritic membrane current $I\left( t \right)$, renamed ‘current input’ in the manuscript to avoid any confusion, responsible for spike generation, is a linearly scaled version of the common synaptic input (CSI) in the single-compartment model with no active conductance considered in this study. With such an approach, the PIC-related nonlinear mechanisms involved into the CSI-I transformation were overlooked, and only later captured with phenomenological parameters such as $IP$, as discussed in the following.

Lines 322 – 368. In this section or in the Discussion you should note that the IP parameter characterizes firing rate saturation that in actual motoneurons is likely to be due to a decrease in driving force for synaptic current flow as the dendrites become more depolarized.

In line with the previous comment, it is now detailed in (L.326-332) that the IP parameter phenomenologically captures the observed saturation in MN firing rate, while this nonlinear behaviour is, in real MNs, due to the PIC-related nonlinear mechanisms occurring in the dendrite compartment that were not modelled in the single-compartment LIF model, which can therefore attain non-physiologically high firing rates for high values of input current $I\left( t \right)$ (and thus of common synaptic drive), if the phenomenological IP parameter is not introduced.

Lines 361 – 367. As was the case for the IP parameter, the change in Rm to induce firing rate hysteresis does not reflect the actual mechanism, which likely arises from hysteresis in the motoneuron’s current-voltage relation (cf. Lee and Heckman, J Neurophys 1998ab).

We thank the reviewer for pointing out that, contrary to the tuning of the $IP$ parameter, which is a purely phenomenological parameter that does not have direct physiological meaning, the gain $k_{R}$ in the $R=\frac{k_{R}}{S^{2.43}}$ relationship is a passive property intrinsic to the MN pool and has a physiological meaning. $k_{R}$ is a passive property independent of the membrane activity and changing its value during the course of the muscle contraction is non-physiological, as now we have discussed in (L.390-397). However, the LIF model proposed in (EQ.7) is not complex enough to model the hysteresis in the motoneuron’s current-voltage relation (Lee and Heckman, J Neurophys 1998ab) that is responsible for the hysteresis between MN recruitment and derecruitment threshold (*Heckman, C. J., & Enoka, R. M. (2012). Motor unit. Comprehensive physiology, 2(4), 2629-2682*.). It is therefore detailed in (L.369-377) that changing the value of $k_{R}$ does not reflect the actual mechanism but is merely a phenomenological interpretation of the MN behaviour at derecruitment where its apparent passive resistance can be considered to increase. In such case, as displayed in Figure 9AB, changing the value of $k_{R}$ provides better predictions of the MN discharging behaviour. We would like to underline that $k_{R}$ is related to MN size as $k_{R}=R\cdot S^{2.43}$, as determined in (Caillet et al., eLife, 2022) and is not exactly the MN specific resistance $R_{m}=R\cdot S$, but rather a constant for the entire MN pool.

Lines 414 – 426. Changing Cm during the course of a contraction may provide a good fit to the firing rate near derecruitment, but it doesn’t provide any insight into the underlying mechanisms governing derecruitment behavior. The only mechanism I can think of to alter the measured capacitance of an area of membrane on this time scale would be to change the area by endo- or exocytosis.

Lines 709 – 723. As mentioned above, changing Cm over the course of contraction doesn’t make a lot of sense, even if it does improve the fit for firing during decreasing drive. Figures 14 and 15 are not necessary and distract from the most important findings of the study presented in Figures 13 and 16, namely the improved prediction of force when the complete simulated MN pool is used to drive the muscle as opposed to just the recorded spike trains.

Line 941. Once again, I cannot think of a mechanism for a time-history-dependent C parameter

These are three important complementary comments on the same topic as the previous $k_{R}$ discussion. As for the value of $R$ ($k_{R}$), $C_{m}$ is a biophysical constant to the MN pool which is independent from the MN membrane activity. Changing its value over the time-course of the contraction is non-physiological and the behaviour of the scaled LIF model cannot provide any insight into the underlying mechanisms governing derecruitment behaviour, as now stated in (L.390-397). As advised, the sections related to this $C_{m}$ sensitivity analysis (including Figures 14 and 15) and its interpretation were therefore removed from the manuscript. In the Discussion section (including L.941), the paragraph attempting to find insights into the MN neuromechanics from the $C_{m}$ sensitivity analysis was also removed.

In this respect, for the recruitment phase and plateau of force, the constant value $C_{m}=1.3\mu F\cdot cm^{2}$ was fixed for all subjects and all MNs and the sensitivity analysis was not performed. This value, which is close to the one proposed by (*Gentet, L. J., Stuart, G. J., & Clements, J. D. (2000). Direct measurement of specific membrane capacitance in neurons. Biophysical journal, 79(1), 314-320*.), comes from the set of relationships provided in our recently published paper (*Caillet, A. H., Phillips, A. T., Farina, D., & Modenese, L. (2022). Mathematical relationships between spinal motoneuron properties. eLife, 11, e76489*) and is consistent with the $C_{m}$ values obtained with the previous sensitivity analysis.

However, as before, considering that the LIF model is not complex enough to describe the hysteresis in the MN’s voltage-current relation that explains the hysteresis in the MN’s current-firing rate relation, the calibration of the $C_{m}$ value at derecruitment was maintained and briefly described in the Methods Section (L.379-389). It is however stressed in the following paragraph (L.390-397) that this approach is only taken for modelling purposes and does not describe the actual MN’s underlying mechanisms. This modelling limitation is now discussed again in the Limitations Section (L.997-1002). To show how well a change in the $C_{m}$ values could capture the hysteresis, we changed Figures 9A and 9B, consistent with a later comment, to show the derecruitment range of the experimental and predicted FIDFs.

Line 405. I’m not aware of any estimate of specific membrane capacitance as high as 10.8 microF/cm2. Please provide a reference.

The range [0.8; 10.8] provided in line 405 of the original manuscript was removed along with the sensitivity analysis section. The 10.8 value was obtained from Table I in (*Albuquerque, E. X., & Thesleff, S. (1968). A comparative study of membrane properties of innervated and chronically denervated fast and slow skeletal muscles of the rat. Acta Physiologica Scandinavica, 73(4), 471-480*.).

Lines 693 – 703. The values of Cm that work best for a single compartment LIF model may have little to do with specific membrane capacitance values for real motoneurons. Variations in time constant and input resistance in real motoneurons are mostly strongly influenced by changes in specific membrane resistivity rather than capacitance or even size (see Gustafsson and Pinter, JPhysiol, 356: 401-431, 1984).

Lines 828 – 841. As mentioned above, the vast differences between a single compartment LIF model and real motoneurons preclude a simple correspondence between LIF parameter values (e.g., size and Cm) and values in actual motoneurons.

Limitations of the approach. The authors should include a section on the uncertain relation between LIF parameters and biophysical mechanisms.

As discussed above, we thank the reviewer for stressing that the single compartment LIF model with no active conductance considered in this study is not suitable for investigating the neurophysiology of individual MNs. In this respect, it was removed from the manuscript any section and sentence inferring the latter, and a subsection of the Limitations Section is now dedicated to the LIF model limitations in describing the true MN neuromechanics beyond capturing its phenomenological firing behaviour (L.975-1033). In this sub-section it is now emphasized that a more realistic MN model is required to investigate the neurophysiology of individual MNs.

We attempted to develop a more realistic formulation for the MN model in (EQ.7) by using the CSI as input to a single-compartment LIF model with active conductance synapse, as suggested in the review from (*Burkitt, A. N. (2006). A review of the integrate-and-fire neuron model: I. Homogeneous synaptic input. Biological cybernetics, 95(1), 1-19*.). In this approach, the main equation of the LIF model is $\frac{dV_{m}}{dt}=\frac{1}{C}\cdot\left( I_{ps}\left( t \right)-g_{R}\cdot V_{m} \right)$ where $I_{ps}$ is the total dendritic membrane current and not the synaptic current, as proposed in our model. $I_{ps}$ is largely influenced by the activity of voltage-dependent dendritic channels that generate intrinsic membrane currents mainly responsible for the MN nonlinear input-output function. Notably, the more the membrane depolarizes, the less sensitive the MN to the synaptic input, which is usually modelled as $I_{ps}\left( t \right)=g(t, CSI\left( t \right))\cdot\left( E_{+}-V_{m} \right)$, where $E_{+}$ is the reversal potential for excitatory channels and $g\left( t \right)$ the time-varying conductance input of the membrane. As in our approach, the CSI is a representation of the net excitatory influx, the activity of the inhibitory channels is not modelled. In a single-compartment approach where the dendritic activity is not described and where all the activity of all the ion channels is lumped into a single representative channel, $g(t, CSI\left( t \right))$ can be described as $g\left( t, CSI\left( t \right) \right)=g_{max}\cdot T\left( t, CSI \right)$, where $g_{max}$ is the maximum active conductance of the membrane and $T\left( t, CSI \right)$ can be either interpreted as the fraction of opened ionic channels in the range $\left[ 0;1 \right]$ or as the synaptic input, as in the review by Burkitt (2006). We tried $T\left( t, CSI \right)=CSI(t)$, i.e., $\frac{dV_{m}}{dt}=\frac{1}{C}\cdot\left( g_{max}\cdot CSI(t)\cdot\left( E_{+}-V_{m} \right)-g_{R}\cdot V_{m} \right)$ using a standard constant value for $E_{+}$ and the same relationships as before linking the passive values of $C$ and $g_{R}=\frac{1}{R}$. With an absolute refractory period of 5ms, we could not make this approach suitable for converting the CSI into a credible firing behaviour with a MN-specific identification of the $g_{max}$ constant parameter. With a $g_{max}$ value set for a physiological discharge rate at recruitment, the $\left( E_{+}-V_{m} \right)$ factor did not bring enough saturation (please refer to Figure 1 below), with small MNs firing still above 60Hz. In line with the work by Powers & Heckman (*Synaptic control of the shape of the motoneuron pool input-output function, J Neurophys, 117(3):1171-1184, 2017*), suggested by the reviewer, the activation of the dendritic Ca channels reaches a saturation with increased conductance input (Figure 5A). In our simpler single-compartment model, this can be interpreted as a saturation in $g$ with increased CSI, in which case $T\left( t, CSI \right)$ represents the degree of opening of the equivalent channel and is a saturating function of the input $CSI\left( t \right)$, the shape and saturating performance of which are MN-specific. We tried for example $T\left( t, CSI \right)=k_{2}\cdot tanh \left( k_{1}*CSI\left( t \right) \right)$ with $k_{2}$ a scaling factor and $k_{1}$ the shape parameter controlling the saturation effect imposed to $CSI\left( t \right)$, which gives $g\left( t, CSI\left( t \right) \right)=\tilde{g_{max}}\cdot tanh \left( k_{1}*CSI\left( t \right) \right).$

Although this approach is more realistic that our LIF model with no active conductances, we believe that its increased complexity is not justified by the current take-home messages of the study that the reviewer encapsulated in his introductory summary. First, contrary to the $IP$ parameter that is conceptually easy to tune against the experimental data and has a convenient phenomenological meaning in default of a physiological meaning, the nonlinearity brought by the $\left( E_{+}-V_{m} \right)$ factor makes it more difficult to reconcile the MN-specific values of $\tilde{g_{max}}$ and $k_{1}$ with the experimental data and requires an additional parameter calibration for these two additional parameters. Moreover, the tanh saturation shape is purely speculative. Finally, despite searching for the best set of values for $S_{MN}$, $\tilde{g_{max}}$ and $k_{1}$, we did not manage to obtain as good predictions of the MN-specific firing activity with the conductance approach as with the current input model. Since the main aim of the revised manuscript is to accurately predict the phenomenological MN spike trains from the experimental CSI, which is achieved as shown in Figures 12 and 13, and not investigating the MN’s detailed working principles, we considered that the current CSI-driven synapse model was the best trade-off between the simplicity of the approach, its performance, and the realistic description of the individual MN dynamics.

Figure 1 : Outputs from the LIF models without (blue dots) and with (orange dots) constant active conductances for a low-threshold representative MN taking $CSI\left( t \right)$ as an input. The IP parameter is set to the actual absolute refractory period (5ms). The x-axis is the level of synaptic input (Left) and time (Right). On the right plot, the experimental FIDF is plotted as a continuous line for comparison.

**Other comments**

Lines 96 - 97. For a consideration of MN-specific nonlinear mechanisms, you should also cite Binder et al. (Nonlinear Input-Output Functions of Motoneurons, Physiology,35(1):31-39,2021) and Powers and Heckman (Synaptic control of the shape of the motoneuron pool input-output function, J Neurophys, 117(3):1171-1184, 2017).

We thank the reviewer for these references which were added to the introduction, and where the tuning of the MN LIF model parameters to phenomenologically capture the nonlinear MN input-output function was discussed.

Lines 334 – 336. How is the IDF trendline calculated? Some kind of smoothing? A polynomial? It would be good to add plot to Figure 2 that shows an example of the IDF trendline superimposed on the instantaneous discharge frequencies.

We thank the reviewer for this comment as the spiking activity is smoothened according to three different approaches in this manuscript, which can be confusing for the reader: low-pass Butterworth filter for the cumulative spike train to obtain the common synaptic input and the muscle neural drive, convolution with Hanning window for the instantaneous discharge frequencies (IDFs) for parameter calibration and model validation, and sixth-order polynomial as in (*Hug, F., Del Vecchio, A., Avrillon, S., Farina, D., & Tucker, K. (2021). Muscles from the same muscle group do not necessarily share common drive: evidence from the human triceps surae. Journal of Applied Physiology, 130(2), 342-354*.) to derive the $IP$ parameter. This was detailed in lines (L.342-343) of the revised manuscript.

Smoothed IDFs obtained with the convolution and polynomial approaches are now displayed in Figure 4C (L.482-489) rather than in Figure 2, to avoid duplicated figures and dedicate Figure 2 to the experimental approach.

In Figure 4B it would be useful to show both the line of best fit and the line of identity.

The line of identity was added to the figure for better visual assessment of the MN’s hysteresis between recruitment and derecruitment thresholds (L.488-490)

In Figure 4C, the fit to the instantaneous discharge frequencies would be clearer if the IDFs were plotted as symbols rather than lines from zero.

As advised by the reviewer, the plot was changed in Figure 4C for a symbol representation of the IDFs.

Please comment on the discrepancy between the time course the low pass filtered CST and the recorded force in Figure 6B. Presumably this reflects under-sampling of low threshold units.
We thank the reviewer for this comment, and we agree that this discrepancy is mostly due to the under-sampling of small units. The consequences of the under-sampling of low-threshold units in the experimental datasets of HDEMG-identified discharging MNs is discussed in detail in the Discussion Section (Benefit 1 L.801 and Limitation 2 L.940), but should be, as advised, mentioned earlier in the manuscript as it impacts the approximation of the common synaptic input (CSI) to the MN pool. In (L.530-535), this discrepancy is identified and related to the coherence values calculated to be below 1.

Figure 9A and B. Why is the ordinate scale for FIDFs from 0 to around 0.25? Shouldn’t it be 0 to 25? The authors should add panels that show how good the fits are as the unit is derecruited.

We thank the reviewer for having identified this issue, which was due to a small bug (mismatched variable) in our code. The comparison of experimental and simulated FIDFs after parameter identification is now reproduced in Figure 9AB for the entire simulation, including the range where the units are being derecruited.

Lines 584 – 590. The surface area in the model is for a single isopotential compartment, whereas motoneurons have extended dendritic structures. The values for surface area in Table 2 and Figure 10 are lower than what is typically seen for total surface area in cat motoneurons which are about three times the values shown here.

This is an interesting comment about a paradox in our single-compartment model. Although the dendritic structure is overlooked in this approach, we still used the total neuron membrane surface area $S$ to determine the MN-specific size-dependent values of $R, C$ and $\tau$ with the relationships in Caillet et al., eLife (2022).

As discussed, the simulations were performed again for this resubmission after the relationships in the aforementioned paper were slightly modified. We now obtain a range of S values that are higher than in the first version of the manuscript: around $\left[ 0.16;0.40 \right]mm^{2}$ for the TA datasets at 35%MVC. Table 2 only reports the minimum value of S in the MN population. To the authors’ knowledge, the $\left[ 0.16;0.40 \right]mm^{2}$ range is slightly lower but in accordance with published data for cat MNs, the largest possible interval found in the literature being $\left[ 0.08;0.76 \right]mm^{2}$:

- Burke et al. 1982 (Fig 2): 0.15-0.48
- Ulfhake & Kellerth 1981 (Fig 11): 0.1-0.75
- Barrett & Crill 1974 (Table 1): 0.08-0.25
- Ulfhake & Kellerth 1984 (Table 1, over 3 muscles): 0.23-0.63
- Ulfhake & Cullheim 1988 (Fig 5): 0.22-0.76
- Gustafsson & Pinter 1984a (Fig.6), calculated: 0.25-0.75

which gives in average among these studies the range $\left[ 0.16;0.57 \right]mm^{2}$.

**Reviewer 2: Kelvin Jones**

Thank you for the opportunity to read and comment on this interesting manuscript. The manuscript presents a modeling framework that combines current “best practise” empirical data collection (decomposition of high-density grid surface electromyography, HDEMG, into discrete spike trains) with a population model of leaky-integrate-&-fire (LIF) neurons. The main contribution is to use the empirical data to constrain parameter choices in the model, and iteratively adjust the model to capture the dynamics of the empirical spike train data. The workflow for this process is new and a unique contribution to the field of modeling in this specialized domain of motor unit physiology. The authors use data from four previous publications to demonstrate a 4-step workflow (Figure 1 and 11) that includes some important validation components that I do not think have been attempted prior to this: 1) the empirical data from individual neurons are assigned rank orders within the population model; 2) the effective neural drive to the pool is calculated from the empirical data; and 3) the predicted discharge statistics from the model are compared to the empirical neuron discharges. The manuscript represents a substantial amount of work and there are many insights to be gained from a careful reading. I offer some general feedback for consideration to strengthen the manuscript and some specific comments below.

We thank the reviewer for this clear summary of our work and its potential impacts, and for appreciating its novelty and the efforts invested in this work. We also thank the reviewer for his very pertinent comments which helped us strengthening the manuscript and better discussing the limitations of the approach. We took care of addressing and answering each of the comments below, systematically referring to the lines of the new version of the manuscript where the changes were made. The changes are highlighted in turquoise in the new manuscript.

# General comments

1. “Computational code is available under request to the authors.” *PLOS Computational Biology*

[*https://journals.plos.org/ploscompbiol/s/submission-guidelines#loc-software-submissions*](https://journals.plos.org/ploscompbiol/s/submission-guidelines#loc-software-submissions) The submission guidelines for PLOS Comp Biology explicitly state that software “must be downloadable anonymously in source code form”, but the manuscript states interested parties must make a request to the authors. My prior reviews for this journal have included links to software on GitHub and I was able to run the modeling software myself during the review process. This permitted a more complete assessment of the PLOS goal that the software “be widely adopted by a broad community of users”. (my paraphrase from the software guidelines) I think the importance of this criteria should be met prior to publication in PLOS Computational Biology and reviewers should have the opportunity to use the software prior to publication. (Of course, I leave this to the discretion of the Editorial staff.)

We thank the reviewer for bringing our attention to the full open-source policy of PLOS Computational Biology. The authors are therefore providing the code open-source via a Github repository. It is for now a private repository, that can be accessed and cloned by the reviewer via this url: https://gitfront.io/r/AHDC/MApvQpDP4YJc/Caillet-et-al-2022-PLOS-Comput-Biol/ . The instructions on how to setup the ad hoc virtual environment necessary to run the scripts without prompting errors, and the description of the aims and contents of the scripts are provided in the repository README file. The authors were careful to comment each code and function in detail to make them as useful and reusable to the reader as possible.

It must be noted that all the simulations were re-run for this resubmission. This was necessary to address the reviewers’ comments and specifically for two reasons associated to the comments. First, $C_{m}$ is now not calibrated anymore in the $\left[ t_{r_{0}};t_{r_{3}} \right]$ range but set to a constant value in all trials, following the reviewer’s comments. Second, the values of the $R,C$ and $\tau$ parameters, based on the mathematical relationships provided in a preprint and now published manuscript (*Caillet, A. H., Phillips, A. T., Farina, D., & Modenese, L. (2022). Mathematical relationships between spinal motoneuron properties. eLife, 11, e76489*), were slightly modified following the reviewing process of that previous manuscript. With these modifications, in the current revised manuscript, the model predictions are strongly in line with the previous results, and overall provide more accurate predictions for Validations 1 and 2. Where necessary, the manuscript text was adapted to reflect the changes.

2. Validation: The manuscript points out two ways in which the modeling workflow was validated (beginning on line 616). First, simulated spike trains are compared to the rank-ordered empirical spike trains that are used to parameterize the model (recognizing the Nr-1 permutations). This is an important step the metrics demonstrate good agreement between the four sets of empirical data and the simulations. However, a key step is missing in this process. All the empirical data that is used to parameterize the model comes from a very specific experimental protocol, isometric trapezoidal force profiles (Fig 2). This is a very useful protocol for data collection, but has low external validity. The manuscript does not provide any evidence that the modeling workflow and parameters generated by fitting to the trapezoidal force profile would generalize to another contraction profile. This would be a very important step to show that the parameters of the model fit in one specific case, generalize to a different contraction trajectory.

We thank the reviewer for this important comment on the applicability of our model, with parameters calibrated on trapezoidal force profile, to other contraction profiles.

To perform such validation, the parameters $IP$ and $S_{MN}$ would be first calibrated against a trapezoidal force profile, from which the distribution of the intrinsic MN parameters $R, C$ and $\tau$ would be obtained. The calibrated distribution of these intrinsic MN parameters would then be reused to scale the $N_{r}^{'}$ MN models that would describe the activity of the $N_{r}^{'}$ MNs identified from another contraction profile. Considering the simple phenomenological approach taken with the $IP$ parameter to capture MN firing rate saturation, the $IP$ parameter would certainly need to be tuned for each contraction profile if the maximum force level is drastically different to that of the trapezoidal profile, although the $IP$ identification process is automated and instantaneous. In a single-compartment LIF model with conductance synapse driven by the CSI (discussed later), it is believed that the synaptic current-to-conductance transformation would similarly need to be re-tuned for new contraction profiles.

We have tried, as suggested by the reviewer in comment #3, to make this methodological manuscript as concise as possible by only focusing on the description, validation and limitations of the four-step workflow, without having an experimental section. This is the reason why we decided to use open-source edited HDEMG data only, to avoid including descriptions of the experimental setup and of the data decomposition and editing processes, which would make the already long Methods Section longer and the manuscript less appealing to readers. To attempt to address comment #2, we performed a literature search looking for open-source edited HDEMG data for various contraction profiles or for trapezoidal contractions up to various force levels (or even for two repeated similar trapezoidal profiles) performed by the same participant. Unfortunately, we could not find any published dataset with such characteristics. As we are currently unable to perform a validation across different contraction profiles, this limitation has been added and discussed in detail in the Limitations Section of the manuscript (L.927-939).

If the reviewer considers that this blind validation for other contraction profiles is a key point, necessary for the validation of the four-step approach before publication, it would be possible for us to perform new HDEMG recordings. It is worth noting that, undertaking this additional experimental data collection would require substantial modification of the manuscript and the inclusion of significant additional material to an already dense manuscript.

3. Line 252 I(t): I liked the care and attention to determining input current to the pool of motor neurons that has empirical origins in the decomposed spike trains. However, the use of current is problematic since synaptic input is a conductance not a current source.

That means as synaptic input increases, the apparent input resistance of the cell decreases and this can give rise to saturation (e.g.

<https://doi.org/10.1152/jn.00850.2016>). Choosing to model this as synaptic conductance would have effects on the IP parameter (Line 322) and may circumvent the need to create ‘saturation’ (line 339) the way it is currently handled. This is a significant limitation (for this reviewer) that could be easily handled by using conductance synaptic input (e.g.

as reviewed in <https://doi.org/10.1007/s00422-006-0068-6>). Attention to this detail seems to be more consistent with the attention to detail in the calculation of effective neural drive (𝑒𝑁𝐷).

To help maintaining a chronological red line in our answers, this comment, previously Comment #5 and now Comment #3, is addressed before the now Comment #4 (previously Comment #3) about the $C_{m}$ parameter.

We thank the reviewer for his appreciation of the care taken in deriving $CSI\left( t \right)$ and for stressing on the limitations of our current single-compartment LIF model with current synapse for providing a realistic description of the underlying neurophysiological mechanisms that explain some of the phenomenological nonlinear MN behaviour such as saturation in MN firing rate with increased synaptic drive. We believe that this is a key comment that we tried to address in the best way possible. We also thank the reviewer for the provided references which helped us addressing this comment.

One of the key contributions of this manuscript is the use of a common synaptic input (CSI), derived from experimental data, to drive a population of MN models. We attempted to use this CSI as input to a LIF model with conductance synapse, as suggested by the reviewer, using for example the provided review from A.N. Burkitt (2006).

In this case, the main equation of the LIF model is $\frac{dV_{m}}{dt}=\frac{1}{C}\cdot\left( I_{ps}\left( t \right)-g_{R}\cdot V_{m} \right)$ where $I_{ps}$ is the total dendritic membrane current and not the synaptic current as proposed in our model. $I_{ps}$ is largely influenced by the activity of voltage-dependent dendritic channels that generate intrinsic membrane currents mainly responsible for the MN nonlinear input-output function. Notably, the more the membrane depolarizes, the less sensitive the MN to the synaptic input, which is usually modelled as $I_{ps}\left( t \right)=g(t, CSI\left( t \right))\cdot\left( E_{+}-V_{m} \right)$ where $E_{+}$ is reversal potential for excitatory channels and $g\left( t \right)$ the time-varying conductance input of the membrane. As in our approach, the CSI is a representation of the net excitatory influx, the activity of the inhibitory channels is not modelled. In a single-compartment approach where the dendritic activity is not described and where all the activity of all the ion channels is lumped into a single representative channel, $g(t, CSI\left( t \right))$ can be described as $g\left( t, CSI\left( t \right) \right)=g_{max}\cdot T\left( t, CSI \right)$ where $g_{max}$ is the maximum active conductance of the membrane and $T\left( t, CSI \right)$ can be either interpreted as the fraction of opened ionic channels in the range $\left[ 0;1 \right]$ or as the synaptic input, as in the review from A.N. Burkitt (2006). We tried $g\left( t, CSI\left( t \right) \right)=g_{max}\cdot CSI(t)$, i.e., $\frac{dV_{m}}{dt}=\frac{1}{C}\cdot\left( g_{max}\cdot CSI(t)\cdot\left( E_{+}-V_{m} \right)-g_{R}\cdot V_{m} \right)$ using a standard constant value for $E_{+}$ and the same relationships as before linking the passive values of $C$ and $g_{R}=\frac{1}{R}$. With an absolute refractory period of 5ms, we could not make this approach suitable for converting the CSI into a credible firing behaviour with a MN-specific identification of the $g_{max}$ constant parameter. With a $g_{max}$ value set for a physiological discharge rate at recruitment, the $\left( E_{+}-V_{m} \right)$ factor did not bring enough saturation, with small MNs firing still above 60Hz (Please refer to Figure 1 below). In line with the work from Powers & Heckman (2017) suggested by the reviewer, the activation of the dendritic Ca channels reaches a saturation with increased conductance input (Figure 5A). In our simpler single-compartment model, this can be interpreted as a saturation in $g$ with increased CSI, in which case $T\left( t, CSI \right)$ represents the degree of opening of the equivalent channel and is a saturating function of the input $CSI\left( t \right)$, the shape and saturating performance of which are MN-specific. We tried for example $T\left( t, CSI \right)=k_{2}\cdot tanh \left( k_{1}*CSI\left( t \right) \right)$ with $k_{2}$ a scaling factor and $k_{1}$ the shape parameter controlling the saturation effect imposed to $CSI\left( t \right)$, which gives $g\left( t, CSI\left( t \right) \right)=\tilde{g_{max}}\cdot tanh \left( k_{1}*CSI\left( t \right) \right).$

Figure 1 : Outputs from the LIF models without (blue dots) and with (orange dots) constant active conductances for a low-threshold representative MN taking $CSI\left( t \right)$ as an input. The IP parameter is set to the actual absolute refractory period (5ms). The x-axis is the level of synaptic input (Left) and time (Right). On the right plot, the experimental FIDF is plotted as a continuous line for comparison.

Although this conductance source approach is more realistic than our current source approach, we believe it has some drawbacks. First, contrary to the $IP$ parameter that is conceptually easy to tune against the experimental data and has a convenient phenomenological meaning in default of a physiological meaning, the nonlinearity brought by the $\left( E_{+}-V_{m} \right)$ factor makes it more difficult to reconcile the MN-specific values of $\tilde{g_{max}}$ and $k_{1}$ with the experimental data and requires an additional calibration for these two additional parameters. Moreover, the tanh saturation shape is purely speculative. Finally, despite searching for the best set of values for $S_{MN}$, $\tilde{g_{max}}$ and $k_{1}$, we did not manage to obtain as good predictions of the MN-specific firing activity with the conductance approach as with the current input model.

Since the main aim of the revised manuscript is to accurately predict the phenomenological MN spike trains from the experimental CSI, which is achieved as shown in Figures 12 and 13, and not investigating the MN’s detailed neuromechanics, we considered that the current CSI-driven synapse model was for now the best trade-off between the simplicity of the approach, its performance, and the realistic description of the individual MN dynamics. We detailed (L.140-143 & L.279-287) that the total dendritic current input $I\left( t \right)$, renamed ‘current input’ in the manuscript to avoid any confusion, and responsible for spike generation, was in this simple model, a scaled version of the CSI. In such case, the PIC-related nonlinear mechanisms involved into the CSI-I transformation were overlooked, and only later captured by the phenomenological $IP$ parameter for the observed saturation in firing rate (L.326-332).

It is then emphasized in the manuscript that a more realistic MN model is required to investigate the neurophysiology of individual MNs. The limitations of the current MN model in describing the true MN neuromechanics beyond capturing its phenomenological firing behaviour are discussed in a new subsection of the Limitations Section (L.975-1033), where other approaches are proposed, such as the synaptic conductance formulation discussed previously.

4. Line 400 Sensitivity analysis - Cm: I have a number of issues with this section. First, specific membrane capacitance is a biophysical property that can be directly measured in a range of tissues (e.g. for measuring excocytosis <https://www.pnas.org/doi/pdf/10.1073/pnas.79.21.6712>, or in blood cells <https://doi.org/10.1098/rsif.2017.0717> ) . It is not a free parameter and the range quoted on 405 is not accurate. Perhaps most relevant to the current manuscript, Cm is a ‘biological constant’ <https://doi.org/10.1016/S0006-3495(00)76293-X> and would best be treated as a fixed value for a pool of motor neurons. The results of fitting Cm (starting line 694) give the expected values, but really add nothing to the value of the manuscript overall. I would suggest removing these sections along with the figures that are based on alterations of specific membrane capacitance (e.g. Figure 14 & 15). A subsequent benefit of removing this material is that it shortens the overall manuscript making it more appealing to readers.

We thank the reviewer for his advice on the sensitivity analysis that was performed on the $C_{m}$ parameter and for the very interesting references. As advised, the sections related to this sensitivity analysis were removed from the Results section. In the Discussion section, the paragraph attempting to find insights into the MN working principles from the $C_{m}$ sensitivity analysis was also removed. For the recruitment phase and plateau of force, the constant value $C_{m}=1.3\mu F\cdot cm^{2}$ has now been fixed for all subjects and all MNs. This value, which is close to the one proposed by Gentet et al. (2000), comes from the set of relationships provided in the recently published manuscript (*Caillet, A. H., Phillips, A. T., Farina, D., & Modenese, L. (2022). Mathematical relationships between spinal motoneuron properties. eLife, 11, e76489*) and is consistent with the $C_{m}$ values obtained with the previous sensitivity analysis. However, considering that the LIF model is not complex enough to describe the hysteresis in the MN’s voltage-current relation that explains the hysteresis in the MN’s current-firing rate relation, the calibration of the $C_{m}$ value at derecruitment was maintained and described in the Methods section (L.379-389). It is however stressed in the next paragraph (L.390-397) that $C_{m}$ is truly constant in actual MNs, and that the approach is purely adopted for modelling purposes. While the change in the $C_{m}$ value may be symptomatic of a change in the MN behaviour at derecruitment, it is non-physiological and does not provide any insights on the true mechanisms responsible for this change. This related modelling limitation is again discussed in the Limitation Section (L.997-1002). As for Comment #3, we preferred improving the accuracy of the predictions rather than opting for a more physiologically realistic model, the predictions of which would be constrained by the model simplicity. To show how well a change in the $C_{m}$ values could capture the hysteresis, we changed Figures 9A and 9B to show the derecruitment range of the experimental and predicted FIDFs.

The range [0.8; 10.8] provided in line 405 was removed with the sensitivity analysis section. The 0.8 value was obtained from (*Sukhorukov, V. L., Arnold, W. M., & Zimmermann, U. (1993). Hypotonically induced changes in the plasma membrane of cultured mammalian cells. The Journal of membrane biology, 132(1), 27-40*) and the 10.8 value in Table I (*Albuquerque, E. X., & Thesleff, S. (1968). A comparative study of membrane properties of innervated and chronically denervated fast and slow skeletal muscles of the rat. Acta Physiologica Scandinavica, 73(4), 471-480*.).

5. Sensitivity analysis - *suggestion*: I think a parameter that would be more interesting to explore in a sensitivity analysis is the number of motor units in the model. The section starting on line 204 indicates that estimates of the numbers of motor units vary from less than 200 to more than 400, and the latter value is chosen. In the anatomical estimate (Feinstein et al. 1955) the authors used old estimates of the proportion of large diameter myelinated axons that were thought to be afferent versus efferent; they assumed 40% of the fibers were afferent and 60% efferent. However, more recent work in the upper limb using improved methods (<https://doi.org/10.1002/ana.25018>, for which Dario Farina is a contributing author) estimate “sensory axons outnumber motor axons by a ratio of at least 9:1”. In Feinstein et al the number of motor axons was estimated as 445 assuming 60% efferents. Recalculation using the 9:1 ratio would give an estimate of about 75 motor units in TA. I think a sensitivity analysis that looked at numbers of motor units ranging from 100 - 400 may have been more interesting compared to the work on specific membrane capacitance mentioned above.

We thank the reviewer for this advice that we applied in the new version of the manuscript. As was already detailed in the first part of the Methods, the number $N$ of motor units in tibialis anterior (TA) or gastrocnemius medialis muscles is under debate, the various identification techniques providing very different orders of magnitude. Consequently, as now described in the Methods section (L.427-431), the four-step workflow was repeated considering MN populations of $N=\left\{ N_{r}, 100, 200, 300, 400 \right\}$ units for the TA muscle, for example. It is concluded (L.706-712 & L.777-782) that the accuracy of the muscle drive predictions obtained with the four-step workflow is not sensitive to $N$. This suggests that the size of the MN population is not a key factor for defining the effective neural drive to the muscle, but the distribution of MN recruitment and firing properties across the considered population is, which is consistent with some conclusions made in the first version of the manuscript on the bias of HDEMG identification towards populations of large units and the undersampling of small early-recruited units. The size of the population may however be of importance for force generation, where the range of motor unit twitch force is shown to be large, up to 90-fold (*Van Cutsem, M., Duchateau, J., & Hainaut, K. (1998). Changes in single motor unit behaviour contribute to the increase in contraction speed after dynamic training in humans. The Journal of physiology, 513(1), 295-305*.). Because the method is not sensitive to $N$, it cannot provide any insight into the real $N$ value in actual MN populations.

The approach proposed by the reviewer to link the $N$ value obtained by Feinstein et al. (1955) with the ratio of sensory axons is very interesting. However, we believe the estimate of 75 units in the TA given by this ratio to be an underestimation of the actual $N$ value, as 67 firing MNs can already be identified at 20-30%MVC, where ~30% of the population is not recruited, with a single multi-channel intramuscular electrode that could not detect the whole muscle volume activity (*Muceli, S., Poppendieck, W., Holobar, A., Gandevia, S., Liebetanz, D., & Farina, D. (2022). ACCURATE DECODING OF THE SPINAL CORD OUTPUT IN HUMANS WITH IMPLANTED HIGH-DENSITY ELECTRODE ARRAYS. bioRxiv*.). While it may be conservative, we therefore kept $N=400$ units in the study.

6. MN-driven muscle model (starting at Line 724): I think that the choice of a Hill-type muscle model (described starting at line 427) is fine for a first iteration. When I read the text and examined Figure 16, I wondered if this step really added to the overall goal of the manuscript. I recognize that adding this was intended to demonstrate an application to a broader potential user base that measures isometric force. My intuition is that comparing the red lines to green lines in Fig 16 may just open the manuscript to criticism given the clear discrepancy in frequency content. Perhaps just the comparison of the blue line to the green is sufficient and an extension to force could come later, combined with models of surface EMG of the single units to compare force and sEMG signals as was done those many years ago in [29].

We thank the reviewer for his insight on the relevance of the neuromuscular application case in the manuscript. We think it is interesting to show the importance of MN pool reconstruction beyond the field of neuroscience in another research area where the interface with the CNS is crucial. In order not to distract the reader from the main message, the final section about neuromuscular modelling has now been reduced by half and moved before the Limitations section, as addressed in your last comment. This study is part of a current larger project on neuromusculoskeletal modelling in which we aim to drive forward MSK simulations with volumetric muscle representations driven at the motor unit level with experimental MN spike trains. The choice of computationally cheap Hill-type models for describing the excitation-contraction dynamics of the motor units (rather than comprehensive muscle models, for example) resides on the high computational cost required for predicting $N$ motor unit forces while maintaining a realistic description of the unit excitation and activation dynamics. Since the first submission, the neuromuscular model has been improved and now builds upon state-of-the-art Hill-type modelling techniques identified in a soon-to-preprint systematic review on Hill-type modelling, and the discrepancy in the frequency content was reduced. A more detailed application of the four-step workflow to neuromuscular modelling is currently detailed in another manuscript in preparation and we wish to keep this application case in the current study to maintain consistency between manuscripts.

# Minor comments

- Page 11, lines 225-227: This was the first instance where in text citations changed from numbers to author name and year of publication. I wasn’t sure if this change was intentional.

We thank the reviewer for having identified this issue, that had arisen after a change in referencing format. The 14 occurrences were consistently modified for a Vancouver format.

- Line 477, Figure 4B: I think the intention in this figure is to show that the data are not well fit to y = x (aka the line of identity). That isn’t immediately clear when looking at the plot because the line of identity is not shown. The regression line (slope 0.9) is fine, but that doesn’t seem to be the point of this figure.

The line of identity was added to the figure for better visual assessment of the MN’s hysteresis between recruitment and derecruitment thresholds (L.488-490)

- I realize that PLOS Computational Biology does not include the guidance to number equations. My preference would be to include numbers, simply because I was doing it myself on my copy of the manuscript. This is a minor issue that I leave to the authors and editors discretion.

We thank the reviewer for this advice that was followed and helped in improving the clarity of the manuscript. The 16 main equations were numbered and referred to in the text.

- Figure 10 C: The presentation of a raster of spike trains is okay, but it might be nice to show instantaneous discharge rate (aka onion skin, e.g.

<https://doi.org/10.1016/j.jbiomech.2014.12.003>) for readers familiar with this way of examining the output behavior of a pool of motor neurons.

We thank the reviewer for this advice and the reference. An additional plot was added to Figure 10, that displays the smoothed instantaneous discharge rate for one out of 20 simulated discharging MNs (L.628-631). The figure was referred to in the Discussion section where the reconstructed discharging MN population is discussed to respect the Onion-Skin scheme (L.786-788).

- Discussion (Limitations, starting Line 871): This was a very extensive section in the discussion and I had to read over it a few times to understand how many discrete instances of limitations were being discussed. Perhaps this could be structurally improved by simply listing in the opening paragraph that X number of limitations will be discussed and then using explicit subheadings: *Limitation 1*, etc. I thought that perhaps

this section was written to preempt reviewers comments and became quite extensive. I think this section could be shortened.

This comment helped us clarifying and re-structuring the Limitations Section, which is now subdivided into:

- Limitation 1 – Model validation
- Limitation 2 – Sensitivity of the method to input HDEMG data
- Limitation 3 – LIF MN modelling
- Limitation 4 – Limited experimental data in the literature

Where possible, the Limitation section was reduced and some limitations of second-order of importance for the main aims of this study were removed.

- Discussion (Line 987 onwards): As stated in general comment #6, I am not sure if the addition of the muscle model helps or hinders the presentation of the framework for developing a pool of spiking neurons to compare with data from HDEMG. This section ends the entire manuscript and as I read it I seemed to be distracted more and more from the key contributions of the paper – rather than reinforcing the important insights gained. I think this could be dropped and replaced with a Conclusion section.

As shortly discussed previously in response to Comment #6, this section was maintained but shortened by half and moved to (L.864-896) before the Limitations Section. A short conclusion (L.1070-1080) was added to the manuscript.
