# Supplementary material for: Estimation of the firing behaviour of a complete motoneuron pool by combining electromyography signal decomposition and realistic motoneuron modelling
Source: PLoS Comput Biol. 2022 Sep 29;18(9):e1010556. doi: 10.1371/journal.pcbi.1010556 (PMC9553065; doi:10.1371/journal.pcbi.1010556)
Supplement: Attachment — Submitted filename: 2022-09-07_RESPONSE TO REVIEWERS.docx [file pcbi.1010556.s003.docx]

RESPONSE TO REVIEWERS – 2^ND^ REVISION

**Reviewer 1: Randall Powers**

‘The authors have done a very thorough job of addressing my concerns. As they point out in their reply, there is a trade-off between the advantages of their LIF models (specifically their simplicity and their prediction accuracy) and the fact that they provide less insight into underlying biophysical mechanisms than more complex Hodgkin-Huxley type models. The benefits and limitations of the LIF approach are now considered in more detail in the revised manuscript. The authors have also removed the use and analysis of a time-varying membrane capacitance which as both Kelvin and I pointed out is quite non-physiological. My only other suggestion for revision is that in section Limitation 3 in the discussion, the authors in might want to mention the use of a two-compartment model (soma and dendrite, e.g., Kim et al. Plos one 9, e95454, 2014) as a potential avenue for future research.’

We thank the reviewer for his help in broadening the future perspectives and research applications of our method. In the previous version of the manuscript, it was already mentioned the possibility of using multiple-compartments Hodgkin-Huxley models for neurophysiological investigations. The reference suggested by the reviewer was added to L.1009 and the text was slightly modified as follows:

‘If a more realistic MN model was considered in lieu of the LIF model, such as multiple-compartments Hodgkin-Huxley-based approaches [128], the four-step workflow would be an adequate tool for testing neurophysiological hypotheses and investigating some mechanisms and properties of the complete MN pool that cannot be directly observed experimentally in conditions of human voluntary contractions. A possible trade-off would be considering a single-compartment LIF model with active conductances […]’
